# Problems with the Concept of “Pest” among the Diversity of Pestiferous Thrips

**DOI:** 10.3390/insects13010061

**Published:** 2022-01-05

**Authors:** Laurence A. Mound, Zhaohong Wang, Élison F. B. Lima, Rita Marullo

**Affiliations:** 1 Australian National Insect Collection, CSIRO, Canberra 2601, Australia; 2 Guangdong Key Laboratory of Animal Conservation and Resource Utilization, Guangdong Public Laboratory of Wild Animal Conservation and Utilization, Institute of Zoology, Guangdong Academy of Science, Guangzhou 510260, China; wangzh@giz.gd.cn; 3 Campus Amílcar Ferreira Sobral, Universidade Federal do Piauí, BR 343, Km 3.5, Floriano 64808-605, PI, Brazil; efblima@ufpi.edu.br; 4 Department of Agricultural Sciences, Università degli Studi Mediterranea di Reggio Calabria, I-81924 Reggio Calabria, Italy; rmarullo@unirc.it

**Keywords:** pest thrips species, Thripinae, *Frankliniella*, *Thrips*, Brazil, China

## Abstract

**Simple Summary:**

The word “pest” can be interpreted in many ways, ranging from something that causes minor personal irritation to something that results in major economic losses. The various insects that are referred to as thrips are used to discuss the question “what is a pest”. Many species of thrips feed on young leaves and developing fruits, and we emphasize that crop loss and reduced financial yield are more significant than mere presence of a thrips on a crop. The diversity in biology among species of thrips is discussed within the context of their respective families and subfamilies, emphasising that pest behaviour is found in relatively few species of the insect Order Thysanoptera.

**Abstract:**

Almost all of the thrips species that are considered pests are members of a single subfamily of Thripidae, the Thripinae, a group that represents less than 30% of the species in the insect Order Thysanoptera. Three of the five major Families of Thysanoptera (Aeolothripidae, Heterothripidae, Melanthripidae) are not known to include any pest species. The Phlaeothripidae that includes more than 50% of the 6300 thrips species listed includes very few that are considered to be pests. Within the Thripidae, the members of the three smaller subfamilies, Panchaetothripinae, Dendrothripinae and Sericothripinae, include remarkably few species that result in serious crop losses. It is only in the subfamily Thripinae, and particularly among species of the *Frankliniella* genus-group and the *Thrips* genus-group that the major thrips species are found, including all but one of the vectors of Orthotospovirus infections. It is argued that the concept of pest is a socio-economic problem, with the pest status of any particular species being dependent on geographical area, cultivation practices, and market expectations as much as the intrinsic biology of any thrips species.

## 1. Introduction

“What is a pest?” seems a particularly simple question. Yet, a simple reply is difficult to construct, because the concept of “pest” is itself a complex socio-economic problem. It involves human perceptions and behaviour, the food production systems as well as the health of our differing societies, and the biogeographic differences in natural and human-made ecosystems. Any definition of “pest” will range from an organism that causes serious economic damage, to an organism that is merely unwelcome at some particular place. This latter definition is by no means unreasonable when we consider the activities of thrips, the members of the insect Order Thysanoptera. These insects commonly display thigmotactic behaviour, with the adults crawling into enclosed spaces such that the maximum area of their body surface is in contact with their surroundings. A typical example is *Limothrips cerealium*, a species known as the Thunder Fly in Britain due to its habit of taking flight from its grass host plants in vast numbers when a summer storm approaches [1]. These thrips swarms may then enter and activate smoke detector fire alarms, causing distress particularly to staff in hospitals. Similar thigmotactic behaviour by *Haplothrips victoriensis* in southern Australia can result in these insects hiding in the central cavity of freshly harvested raspberries; this thrips does not feed on or damage the crop, but its presence within the fruit is clearly unwelcome. Similarly, the bean thrips of California, *Caliothrips fasciatus*, crawls into the navels of oranges where it is regarded as a quarantine risk by countries importing the fruit [2]. In some warmer countries the leaves of *Ficus* trees are often galled by *Gynaikothrips ficorum*. These large thrips fly around and sometimes fall into a glass of wine or beer held by someone relaxing beneath the shady trees—that person would certainly consider that particular thrips a pest. Thus, the statement by Lewis [3] that “Several hundred species of thrips are pests” depends on how the word “pest” is interpreted. For the purposes of this article, we focus our concept of “pest” on those thrips species for which we have reason to believe they are associated repeatedly with serious damage to cultivated plants (Table 1). 

We have two objectives here. One is a factual account of the systematic relationships amongst the various Thysanoptera species that have been reported to be pestiferous in some part of the world. In doing this we emphasize the relatively close evolutionary relationships amongst thrips species that are considered to be pests; most are members of just one subfamily, the Thripinae. The other objective is more contentious. We examine the unpredictability of thrips species as pests, both in time and space, emphasising that different parts of the world have different climates and crops, with different societies having differing expectations. For this we have tabulated the thrips considered to be pests in Brazil and China (Table 2 and Table 3), two widely differing countries with diversified crop production systems. The first of these essentially represents a single biogeographical, Neotropical, entity. In contrast, China is biogeographically complex, from the Palaearctic of Heilongjiang, to the Oriental fauna of Hainan and Yunnan, with the Province of Xinjiang in the west sharing faunal elements with Iran.

## 2. Assessing the Pest Status of Thrips

**Figure 1:**
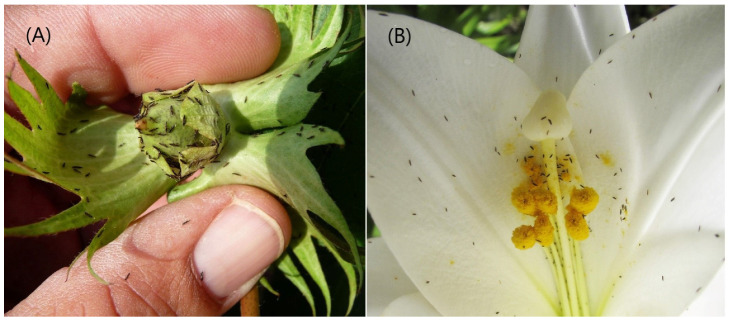
Aggregations of adult thrips. (**A**) Black Plague Thrips (*Haplothrips froggatti*) on cotton bud. (**B**) *Thrips parvispinus* on garden Lily flower.

The published literature about thrips commonly involves an assumption that any thrips found on a cultivated plant will be a pest. This assumption can occur even in the absence of any evidence of damage, let alone crop loss or economic impact. One example, also mentioned below, is the Black Plague thrips in Australia (Figure 1A) that disperses from its grass host plants as these dry in summer; vast numbers then land on irrigated crops where they do not breed. Mass flights also occur in *Thrips australis*, a species that breeds primarily in the flowers of *Eucalyptus* trees that commonly have mass-flowering periods. All of the flowers on a single tree thus die within a short period, and the adult thrips then disperse in very large numbers, seeking shelter in the flowers of many different surrounding plants but without breeding in them. This phenomenon of massed flights of adults occurs in other thrips species but again, it is not necessarily associated with any damage to the plants (Figure 1B). 

Even when some cultivated plant shows symptoms of thrips feeding damage, the culprit pest is not necessarily the most visible or abundant species. For example, a severely damaged crop of *Solanum melongena* in Brazil was observed to bear large numbers of the highly visible and dark species, *Caliothrips phaseoli*, but the leaf damage to the plants was due to small numbers of the small pale species, *Thrips palmi* [4]. Additionally, economically significant damage can occur on some sensitive fruit crops with remarkably low thrips populations. Even a single larva of *Frankliniella occidentalis* feeding under the sepals on a young nectarine (*Prunus persica*) may cause undesirable skin blemishes as that fruit expands. 

A further confusion for any simple definition of “pest” is due to the opportunistic behaviour that is widespread amongst Thysanoptera [5], such that a known crop pest may behave as an effective predator, constraining populations of leaf mite pests [6,7]. Moreover, the pest status of a species may change with time, such that the European species, *Taeniothrips inconsequens*, was an important pest of pear trees in California early in the 20th century, but is now of little importance in that area, although later in the century it became a major pest causing defoliation of sugar maple trees in northeastern USA [8]. Similarly, *Thrips calcaratus* has been responsible for damaging the leaves of *Tilia* trees in eastern USA [9], but populations in its native habitats in Europe are small and often localized [10]. 

Any attempt at a broad definition of pest must consider that “pest” is not an essential attribute of any thrips species. A species that is associated with crop damage at one site does not necessarily cause damage at some other site. Additionally, “crop damage” itself can vary from trivial markings on leaves to crop failure and economic losses. For example, *Anaphothrips obscurus* and *A. sudanensis* sometimes occur at some localities in sufficiently large numbers to cause visible leaf damage to cereal crops, but more commonly the populations of these thrips are inconspicuous and not associated with any visible damage. Other thrips species that are listed as pests have restricted distributions and thus have a relatively local economic impact; for example. *Bradinothrips musae* on banana trees in Brazil [11], or *Ceratothripoides claratris* as a virus vector on tomatoes in parts of southeast Asia [12]. At the opposite extreme there are polyphagous species such as *Frankliniella occidentalis* and *Thrips tabaci* whose feeding and virus vectoring have come to involve serious economic losses worldwide [13]. It is among these, and similar highly polyphagous species, that the most important thrips pests have developed. 

## 3. The Diversity of Pest Thysanoptera 

About 6300 thrips species are currently recognized [14], and these are considered to be members of one or other of the two Thysanoptera Suborders, the Terebrantia and the Tubulifera. The latter comprises 65% of all Thysanoptera species, and these are placed in the single family Phlaeothripidae. Remarkably few of the 3800 species in this family can be considered pests, with the majority of the species feeding only on fungi [15] on dead leaves and branches; these species may even be beneficial in facilitating nutrient recycling. The remaining Phlaeothripidae species are found mainly feeding on green leaves where they often cause leaf distortions or even galls; relatively few species live and breed in flowers. Typical leaf-galling thrips are the *Gynaikothrips* species on the leaves of *Ficus* species that are widely planted as amenity trees in tropical and subtropical areas [16]. However, there seems to be no demonstration that this inhibits the growth of infested trees. Similar leaf damage is caused on persimmon trees (*Diospyros kaki*) in Japan by *Ponticulothrips diospyrosi* [17]; in southern Europe, *Liothrips oleae* is known to cause leaf distortions on olive trees [18]; several species of *Liothrips* are associated with similar damage to *Piper* vines, including commercial pepper crops, between southern India and northern Australia [19]. In South America, *Holopothrips fulvus* has been reported damaging the foliage of Cashew trees (*Anacardium occidentale*) [20], and *Pseudophilothrips adisi* is considered the key pest on cultivated Guarana (*Paullinia cupana*) [21]. One rare demonstration of actual damage to the growth of a tree by a leaf-feeding phlaeothripid was provided by [22]. These authors recorded an experiment with almost 50% reduction in tree height to *Polyalthia longifolia*, a widespread Asian amenity tree, due to infestation by the leaf-galling species *Crotonothrips polyalthiae*. Even more severe damage, leading to the death of the endemic plant *Myoporum sandwichense*, has been recorded on Hawaii by the introduced Australian phlaeothripid *Klambothrips myopori* [23]. In contrast, few Phlaeothripidae breed in flowers, although some that breed in the florets of Poaceae can occur in very large numbers. One such species, *Haplothrips froggatti*, is known as the Black Plague thrips in Australia because vast numbers of adults fly off as the grass heads dry out, and these thrips may then land on and seek shelter on irrigated plants on which they neither feed nor breed (Figure 1A). 

The Thysanoptera Suborder Terebrantia comprises eight families [14], and the majority of pestiferous thrips are members of the single family Thripidae. It is only within this family that the vectors of Orthotospoviruses are known, and these vector species are the thrips that cause the greatest economic damage worldwide. Indeed, the major thrips pests are all members of the Thripinae, the largest of the four subfamilies recognized in the Thripidae. Each of the other three subfamilies, Panchaetothripinae, Dendrothripinae and Sericothripinae, includes a few pest thrips, but with only one species known as a virus vector. The discussion below about pest thrips is organized around these four subfamilies. 

## 4. Pestiferous Panchaetothripinae

With the exception of *Caliothrips* species, the species of this subfamily breed mainly on older leaves rather than on newly emerged leaves, and not in flowers. They are thus very different in behaviour and effect from most pest species of Thripinae, such as *Scirtothrips* species that feed particularly on young, developing tissues. The Panchaetothripinae comprises about 150 species, of which the most well-known is the Greenhouse Thrips, *Heliothrips haemorrhoidalis*. First described from Europe, but native to South America [11,24], this thrips is now found throughout the world. In cool climates, it is usually found only in sheltered conditions, but in warmer climates it breeds readily out of doors. All life-stages of this species live on mature leaves, and affected leaves usually bear dark spots of faecal material that have been exuded by the larvae. It has been found breeding on a wide range of plant species, but is particularly associated with hard leaves, such as those of *Arbutus unedo* or *Camellia sinensis*. However, ferns with relatively soft fronds sometimes bear large populations, particularly when water-stressed; this thrips was reported recently from strawberries in Brazil [25]. 

Several species of the genus *Helionothrips* are similar in appearance and biology to *Heliothrips* species, with *H. aino* forming large populations on Taro (*Colocasia esculenta*), and *H. annosus* similarly on *Cinnamomum burmanii*, with the leaves of these plants often showing white patches. The red-banded cacao thrips, *Selenothrips rubrocinctus*, also *Retithrips syriacus*, commonly damage the leaves of various plants in tropical countries, ranging from cocoa to roses. In the greenhouses of temperate areas *Parthenothrips dracaenae* sometimes causes similar problems. However, it is unusual for any of these species to be associated with crop yield reduction. The genus *Hercinothrips* includes at least three species that are associated with damaged leaves: *H. bicinctus* and *H. femoralis* both develop large populations on the older leaves of a wide range of plants; and *H. dimidiatus* has recently become a pest in southern Europe on decorative *Aloe* plants [26]. The genus *Caliothrips* includes several species that are associated with the leaves of Poaceae including Sugar Cane, for example *C. striatopterus*. Other *Caliothrips* species are reported to damage crop plants, such as *C. impurus* and *C. sudanensis* on seedling cotton in Africa. As mentioned above, *C. fasciatus* is considered a quarantine risk when found hiding in imported navel oranges. Several other species of this subfamily are sometimes found irregularly in association with leaf damage (Table 1), but they are rarely reported to be of economic importance except locally. 

## 5. Pestiferous Dendrothripinae

Just over 100 species are known in this group, and they all feed and breed primarily on mature leaves rather than on young newly emerged leaves. Almost any of them may, at times, be associated with feeding symptoms, but very few are known to cause serious damage. *Dendrothrips ornatus* sometimes causes pale markings on the leaves of Privet (*Ligustrum vulgare*) in Europe, as well as similar symptoms on Lilac (*Syringa vulgaris*), a related member of the Oleaceae [27], in northern China. However, the only crop reported to be seriously damaged by a *Dendrothrips* species is *Camellia sinensis* in China, where *D. minowai* usually develops large populations. Two species of *Pseudodendrothrips* are also recorded in association with leaf damage: *P. mori* can be particularly serious on mulberry (*Morus alba*) in dry climates [28], but *P. stuardoi* seems to be less damaging on fig trees (*Ficus carica*) [29]. In South America, *Leucothrips* species are sometimes reported in association with leaf damage to capsicum crops and also to crops of *Sechium edule* [30].

## 6. Pestiferous Sericothripinae

This subfamily currently comprises only three genera, with a total of 170 species [14], but only a few species in the genus *Neohydatothrips* have been associated with damage to any plants. The most important one, *N. variabilis*, is the main vector of soybean vein necrosis virus across much of North America [31]. This is the only Orthotospovirus known to be vectored by any thrips species that is not a member of the Thripinae. Considering the widespread cultivation around the world of soybean (*Glycine max*), this combination of thrips and virus has the potential of becoming of major economic importance. In Colombia, *N. burungae* (but under the name *signifer*) has been reported as damaging Passion fruit vines (*Passiflora edulis*) [32], and in southern China, *N. flavicingulus* has been found in large numbers damaging the leaves of Camphor trees (*Cinnamomum camphora*). The marigold thrips, *N. samayunkur*, occurs widely around the world inducing damage to the leaves and flowers of garden plants in the genus *Tagetes* [33,34]. The only other sericothripine species that has an impact on the human economy is *Sericothrips staphylinus*, a species that has been used in the biological control of Gorse (*Ulex europea*), an invasive weed in several countries [35]. 

## 7. Pestiferous Thripinae

**Figure 2:**
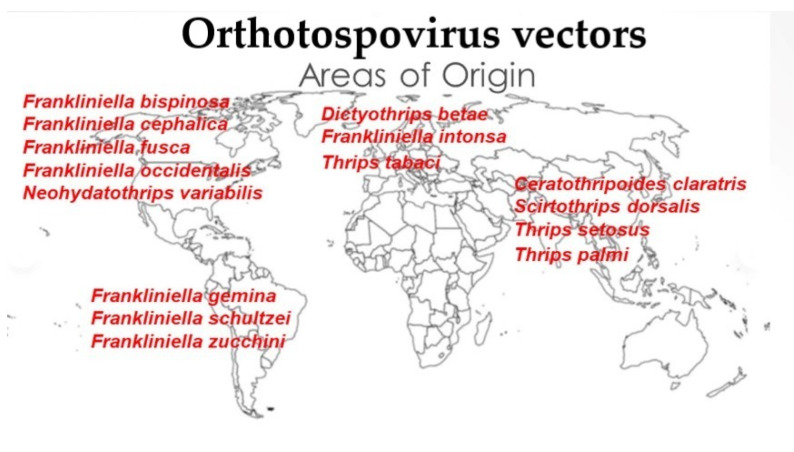
Geographical origins of Orthotospovirus vector species.

Before considering the pest status among the 1800 known species in this subfamily it is important to emphasise that less than 1% of these species are recorded as vectors of the serious crop diseases known as Orthotospoviruses [36]. With the exception of *Neohydatothrips variabilis* in the Sericothripinae, the other 14 species recorded as vectors of this group of plant viruses are all members of the Thripinae, and these species are members of five different genera (Figure 2). Eight vector species are members of the genus *Frankliniella*: three are members of the genus *Thrips*; the remaining three are each placed in different unrelated genera. This suggests that the association between thrips and the various Orthotospovirus species has arisen independently several times and on different continents. Zhang et al. [36], in recognizing 30 species of Orthotospovirus stated that 17 of these are reported from China (including undescribed species). However, no known vector species is endemic to China, although *Thrips palmi* is possibly native in the tropical southwest of that country. In contrast, eight of the 15 thrips vector species are originally from the American continent, with five from North America, and three from South America (Figure 2). 

### 7.1. Thripinae Pest Species in Genus-Groups

The evolutionary relationships among the 230 genera recognized in this subfamily remain far from clear, with only a few major supra-generic groupings reasonably diagnosed [37]. Among the 90 listed species of Thripinae in Table 1 there are two major groups: *Frankliniella* species mainly from the New World; *Thrips* and related genera mainly from the Old World. Of the smaller groups, the *Megalurothrips* group is almost entirely Old World in its distribution, as is the *Mycterothrips* group. In contrast, *Scirtothrips* and its relatives are essentially pantropical, and the *Anaphothrips*, *Chirothrips* and *Taeniothrips* groups also all have native species in widely separated parts of the world. The range of pest species within each of these groups is here considered separately. 

#### 7.1.1. *Frankliniella* Genus-Group

This genus-group includes *Frankliniella*, with almost 240 species, plus six small genera [38] that comprise a total of 33 species. These small genera are all from the Old World, and, apart *Kakothrips pisivorus* that was described originally as a pest of pea crops in Britain, none of the included species are considered pests. In contrast, *Frankliniella* is almost entirely New World in origin, with only six species native to the Palaearctic [39]. Of these six species only *F. intonsa* is seriously pestiferous at times, whereas amongst the many New World species there are several that are associated with damage to cultivated plants. 

Some of these New World *Frankliniella* pest species are restricted in their host range, or at least in the range of plants on which they have been reported to cause damage. For example, *F. melanommata*, sometimes causes extensive damage to crops of cassava (*Manihot utilissima*) in parts of South America [40], and in various countries the Poaceae-living species, *F. tenuicornis* and *F. williamsi*, can develop large populations on the leaves of maize (*Zea mays*) [39], although usually without serious yield reduction. Two species that possibly have a wider host range, *F. brevicaulis* and *F. parvula*, are particularly associated with damage to bananas (*Musa sativa*), and both are also commonly associated with cocoa trees (*Theobroma cacao*) [40]. A few common species occur on many different plant species including crops and garden ornamentals (*F. gardeniae*, *F. insularis*, *F. tritici*), but are not usually considered serious pests. Then, there are eight highly polyphagous pest species of *Frankliniella*, of which seven originate from the American continent (Table 2). 

Unfortunately, species recognition in this genus continues to pose considerable problems. For example, *F. gemina* cannot be distinguished satisfactorily from *F. zucchini* [41], despite them both being considered Orthotospovirus vectors. Even greater confusion exists with the worldwide pest, *F. schultzei*, within which molecular studies have distinguished a complex of sibling species [42]. However, no evidence has been produced of any consistent biological differences between these siblings, including their ability to transmit Orthotospoviruses. As a result, for the purposes of practical horticulture and agriculture, *F. schultzei* continues to be treated as a single entity. 

*F. occidentalis* is the thrips species that has become of greatest economic importance in the past 50 years [43]. Individuals taken from crops around the world are remarkably uniform in their colour and structure, and can be identified from the CO1 gene [44]. However, the species is considered to have come from western North America. In the mountains of California, populations of either yellow or dark brown forms have been collected in the absence of the bicoloured pest form that is common on crops in the California Central Valley, but there has been only one study of molecular variation among Californian *F. occidentalis* populations. This indicated the presence of two sibling species [45], one of which occurs worldwide and the second (the Lupin strain) known from China and New Zealand as well as California. However, this study did not include the native colour variants from the mountains of western USA. Breeding experiments in California [46] indicated that the colour forms segregated according to Mendelian expectations, but those results do not accord with the fact that dark forms of this species occur in various parts of the world that are subject to low temperatures, even in winter in parts of eastern Australia. Despite this species being so well-known [47], further studies on the native populations of *F. occidentalis* in western USA, including experimental rearing together with associated molecular work, could yield a better understanding of the origin of this remarkable pest. 

#### 7.1.2. *Thrips* Genus-Group

About 15 genera are considered members of this genus-group, with 300 species in the genus *Thrips*. Of the other 14 genera, eight of them each includes a single species, and the remaining six genera comprise a total of 67 species. Apart from members of *Thrips* genus, the only other members of this genus-group that involve pest species are species of three Old World genera associated with Poaceae, *Bolacothrips*, *Fulmekiola* and *Stenchaetothrips*. The 13 species listed under *Bolacothrips* are of minor importance, but *F. serrata* is sometimes considered an important pest on young sugar cane. Amongst the 42 species of *Stenchaetothrips* several are associated with leaf damage on cereal crops or even on decorative species of bamboo [48]. One of them, the Rice Thrips, *S. biformis*, used to be a serious pest in beds of rice seedlings, but it is usually of limited importance on established rice crops that are grown using modern cultivation techniques. 

Among the many species of *Thrips* genus several have been recorded as damaging plants, including a few species that have a restricted host range. Feeding by the Gladiolus Thrips, *T. simplex*, can cause linear markings on the leaves and flowers of cultivated *Gladiolus* plants as well as a few related Iridaceae [49]. The Linden Thrips, *T. calcaratus*, is largely restricted to the leaves of *Tilia* species [9], the Hop-flower thrips, *T. albopilosus*, is sometimes abundant on *Humulus lupulus* in Europe, and *T. alliorum* is sometimes a problem on chives and onions in China [50]. It is amongst the polyphagous species of the genus that serious pests are found, although wide host range is not closely correlated with pest status. For example, *T. hawaiiensis* is often abundant in tropical and subtropical countries and can be found in the flowers of many different plants; but high numbers of adults are not always associated with serious crop damage or yield reduction. Moreover, in Malaysia this thrips is considered beneficial in being the main pollinator on plantations of Oil Palm, *Elaeis guineensis* [51]. Similarly. *T. parvispinus* is generally a harmless flower thrips, but at some localities on particular plants, such as in Hawaii on pawpaw (*Carica papaya*), it can cause economic problems [52]. 

This situation of unpredictable pest status occurs with several of the *Thrips* species listed here (Table 1). Thus *T. obscuratus* is an abundant species that is widespread in New Zealand on many native plants [53], although locally it can be an important pest in fruit orchards. Similarly, although the highly polyphagous Australian Plague Thrips, *T. imaginis*, was considered an orchard pest early in the 20th century it is currently not usually associated with extensive crop damage. This is not the only thrips species in which behaviour has changed over time. One polyphagous species native to Japan, *T. setosus*, is typically associated with crops of Solanaceae in that country [54], but in recent years has become of quarantine significance, being found on the leaves of *Hydrangea* plants imported into Europe. 

**Figure 3:**
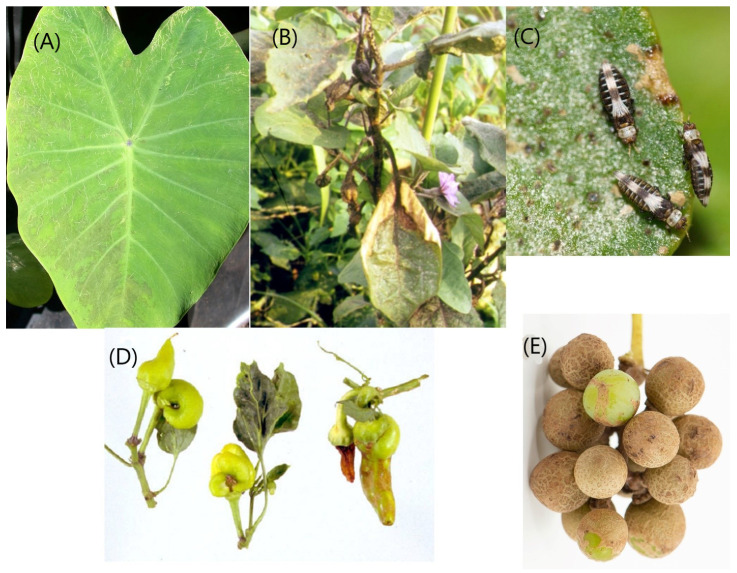
Feeding damage by thrips. (**A**) *Biltothrips minutus* markings on leaf of Taro (*Colocasia esculenta*). (**B**) *Thrips palmi* damage to aubergine crop (*Solanum melongena*). (**C**) *Dendrothrips ornatus* on *Ligustrum* leaf. (**D**) Damage to *Capsicum* fruits by *Franklliniella occidentalis* and Orthotospovirus. (**E**) Surface damage to grapes (*Vitis vinifera*) by *Scirtothrips dorsalis*.

Worldwide, the two most important pest species in the genus *Thrips* are *T. palmi* and *T. tabaci*. Both of these are Orthotospovirus vectors, and although flower-living they both cause serious feeding damage on the leaves of some plants that they attack. The Melon Thrips, *T. palmi*, is a major pest in tropical countries [55], sometimes almost defoliating crops of Eggplant (*Solanum melongena*) (Figure 3B), although it also causes serious damage to crops of other Cucurbitaceae related to melons. Similarly, the Onion Thrips, *T. tabaci*, is highly polyphagous and particularly known as a pest of species in the genus *Allium*. However, it causes considerable damage to *Brassica* crops and is sometimes associated with distantly related plant species such as wheat (*Triticum*). However, *T. tabaci* shares with *Frankliniella schultzei* the ability to be a beneficial insect when it acts as a predator on mites, including Eriophyidae [56].

#### 7.1.3. *Megalurothrips* Genus-Group

The three major genera that comprise this group all involve species that breed mainly in the flowers of Fabaceae. Species in two of them, *Odontothrips* and the Australian genus *Odontothripiella*, are rarely associated with crop plants, whereas several species of *Megalurothrips* are important as pests of bean crops in tropical and subtropical countries [57,58,59]. Two further genera that are possibly related to this genus-group are *Ceratothripoides* and *Pezothrips* [37]. The first includes *C. claratris* that is an Orthotospovirus vector on tomato plants in Asia [12], and the second includes an Australian species, *P. kellyanus*, that causes damage to citrus fruits in Australia, New Zealand and the European Mediterranean area [60]. 

#### 7.1.4. *Scirtothrips* Genus-Group

Although nine genera are placed in this group [61], totalling almost 120 species from various tropical countries, more than 100 of these species are members of the genus *Scirtothrips*. Adults and larvae of species in this genus feed on young, rapidly growing, plant tissues. As a result, they not only cause damage and distortion to newly emerging leaves, but also cause serious economic damage to the fruits of *Citrus* and avocado by feeding on these in the earliest stages of fruit development [62]. Feeding on young citrus fruits by *S. aurantii* in South Africa, and *S. citri* in California, leads to similar symptoms of a ring of scar tissue around the fruit attachment point. Similarly, *S. perseae* feeds on the surface of young avocado fruits in California leading to extensive surface damage as each fruit expands, or even limiting the growth of a fruit. Two further *Scirtothrips* species cause leaf damage in particular regions, *S. manihoti* on cassava plants in South America [63], and *S. mangiferae* on mango plants in western Asia [64]. In contrast, the Asian species, *S. dorsalis*, is important as a vector of Orthotospoviruses. This species is known as the Chilli Thrips due to the damage caused to *Capsicum* crops in India, but it is also a pest of Tea (*Camellia sinensis*) and of Lotus (*Nelumbo nucifera*) [65]. In recent years it has become well-established as an invader in the Caribbean region and Florida [66]. One thrips in a closely related genus, *Drepanothrips reuteri*, is sometimes a pest on the leaves of vines (*Vitis vinifera*) in southern Europe. Similarly, the tiny species, *Biltothrips minutus*, has been found causing linear markings on the leaves of Taro (*Colocasia esculenta*) (Figure 3A) as well as on cassava (*Manihot esculenta*) and some other crops but without obvious crop loss [23]. 

#### 7.1.5. *Taeniothrips* Genus-Group

This is a weakly diagnosed group of nine small genera, mainly from the Old World [67]. Introduced to North America, *Taeniothrips inconsequens* was a serious pest of pear crops (*Pyrus* spp.) in California early in the 20th century, but late in that century it was responsible for extensive defoliation of Sugar Maple trees (*Acer saccharum*) in northeastern USA [8]. The Cardamom thrips, *Sciothrips cardamomi*, is an Asian species apparently specific to the flowers of this crop (*Elettaria cardamomum*) on which it is a minor pest in Central America. Of the other species in this genus-group, adults of *Tenothrips frici* are often found on different plants whilst breeding particularly in the flowers of several weedy Asteraceae, and *Ctenothrips kwanzanensis* has also been taken from many different plants and is widespread in the mountains of China and Japan, but neither of these species is associated with serious crop damage. 

#### 7.1.6. *Mycterothrips* Genus-Group

This group comprises about five genera of leaf-feeding species mainly from the Old World [68], but relationships among these genera have been questioned in a more recent phylogenetic study [37]. *Mycterothrips glycines* is closely associated with crops of soy beans (*Glycine max*) on which large populations may cause leaf damage in parts of Asia, but the other members of this genus are of little economic significance. Species of *Dichromothrips*, the Old World orchid thrips, feed specifically on various Orchidaceae and sometimes cause visible damage to the flowers of these valuable plants in cultivation. One little-known species described from coffee in Kenya over 100 years ago, *Diarthrothrips coffeae*, has more recently been reported as a pest of this crop in southern Ethiopia [69]. 

#### 7.1.7. *Anaphothrips* Genus-Group

A total of 20 genera worldwide is included in this group, although there are good reasons for doubting if all of these are closely related [70]. None of the included species is considered a major crop pest, but large populations of some species may induce leaf damage involving linear markings on the leaves of cereal and grass crops. *Anaphothrips obscurus* is particularly common on wheat crops in relatively cool areas including India and parts of North America [71], whereas *A. sudanensis* is a tropical species that breeds on various Poaceae including sugar cane. Similarly, large populations of the wingless European *Aptinothrips rufus* may create problems in some grass crops that are being grown for seed, and in Brazil, *Psydrothrips kewi* has been reported as damaging crops of Calla lilies [72]. *Apterothrips apteris* has been found on the leaves of plants as different as garlic and lucerne, but it is not usually considered a pest, although a single potted Asteraceae plant was found dead through the feeding by a large population [73]. The only Orthotospovirus vector species in this group is a central European species, *Dictyothrips betae*, that is recorded as a virus vector on weedy species of the genus *Polygonum* in Italy [74]. 

#### 7.1.8. *Chirothrips* Genus-Group

The members of this group all feed on Poaceae, with *Limothrips* species breeding on the leaves of grasses, and species of *Arorathrips* and *Chirothrips* breeding in the florets of grasses [75]. The European species *L. cerealium* has been considered a pest of grass production in parts of North America. However, it plays a more significant role in human society through its habit of over-wintering in confined spaces, such as the back of framed pictures and computer discs, but particularly in smoke alarms which it can trigger in late summer [1]. The 16 species of *Arorathrips* are all New World in origin [76], although *A. mexicanus* is now found worldwide in tropical areas and has sometimes been taken in the flowering heads of sugar cane. In contrast, the 42 species of *Chirothrips* are from many different parts of the world. As with the species of *Arorathrips*, the eggs of these thrips are laid within a grass floret where each larva develops at the expense of an individual grass “seed”. *C. manicatus* has been known as the Timothy Thrips and has been considered a pest in New Zealand in the production of Cocksfoot (*Dactylis glomerata*). 

#### 7.1.9. Ungrouped Genera

Although a wide range of genera is considered here, few of them involve species of any serious economic importance. One species from eastern North America, *Echinothrips americanus*, variously known as the Impatiens Thrips or the Poinsettia Thrips, has caused considerable economic problems through damaging the leaves of a wide range of plants in greenhouses, including *Capsicum* crops [77]. In parts of Asia on the leaves of sweet potato (*Ipomoea batatas*) both *Bathrips melanicornis* and *Dendrothripoides innoxius* are common, but despite the leaf damage there is little evidence of a serious effect on crop yields. On crops of coffee in Asia, between India and Timor Leste, *Euphysothrips subramanii* can be common on the leaves, but this species apparently feeds on the fungus, coffee leaf rust (*Hemileia vastatrix*), rather than on the leaf tissues [78]. Similarly, *Foliothrips traegardhi* is widely reported across Africa and India and is often listed as a pest on a wide range of plants, but without any published information about crop loss. This is possibly another example of a species that, because it sometimes occurs in large numbers, is regarded as a pest but with little evidence of any effect on crop yields. In contrast, *Enneothrips flavens* is sometimes a serious pest causing extensive foliar damage and yield reduction to groundnut crops in parts of South America [79]. Several species in the genera *Chaetanaphothrips* and *Danothrips* are known to cause leaf damage to a range of unrelated plants, such that *C. signipennis* is sometimes called the banana rust thrips, and *C. orchidii* has been called the Anthurium thrips and also the Orchid thrips, despite being known as a pest of Citrus in Florida [80]. 

## 8. Discussion

Given the right conditions, any phytophagous Thysanoptera species may develop locally a particularly large population and cause visible damage to some part of a plant. However, there is a considerable economic difference between the markings on a few leaves of *Ligustrum* due to the feeding of *Dendrothrips ornatus*, and the defoliation of a valuable crop of *Solanum melongena* by a population of *Thrips palmi*. It is the loss of income to a cultivator, whether through yield reduction or through quality reduction, that is the ultimate criterion of pest status. However, this is not a simple quality of a particular thrips species; it involves many variables other than the variation in the biology and behaviour of a pest thrips across its range. *Thrips tabaci* can be a serious pest on onions, but it occurs widely across Australia (and other places) on many different plant species on which it is rarely any problem. Similarly, *Danothrips trifasciatus*, *Chaetanaphothrips orchidii*, and *C. signipennis* are all recorded in various parts of the world as damaging plants as different as bananas, citrus and orchids, but each of these species is more commonly found in low numbers. In Brazil, *Frankliniella schultzei* is considered a major pest, whereas in China this species is not listed as a serious pest. Moreover, at any one site pest populations can be transient. For example, in March 2014 extensive leaf damage by *C. orchidii* to *Commelina cyanea* was observed on Norfolk Island, but six months later that same stand of plants had very few *C. orchidii* but large numbers of *Hercinothrips bicinctus* [73]. Why thrips populations should be so unstable is a question that is beyond the scope of this article, but it seems to be inherent among Thysanoptera. 

Thrips that breed only on older leaves are least likely to severely affect the yield of a crop, although the unsightly cosmetic damage on leaves soiled by *Heliothrips* faecal droplets can have financial implications. In contrast, all *Scirtothrips* and some *Thrips* species feed on young tissues. These may, at relatively low populations, inflict serious damage to emerging leaves and developing fruits with resultant economic losses. Additionally, presence of a suitable thrips vector together with an Orthotospovirus can result in total loss of income to a cultivator. The unpredictability of thrips species is reflected not just in the diversity of plants that they might attack, but also in the time and place of such attacks, and the population size. In publishing an account of the many species of *Scirtothrips* known from Australia, Hoddle and Mound [81] stated that no specimens of the Oriental pest species, *S. dorsalis*, had been seen on this continent from south of Brisbane. However, in 2020 large populations of this thrips were reported near Perth in Western Australia causing considerable damage to rose plants and to the leaves and fruit on grape vines (Figure 3E). 

Population size and economic damage by thrips species are thus essentially unpredictable in time, space and crop. Different parts of the world often cultivate different crops and also have a different native thrips fauna, and as a result the species recorded as pests also differ, with Brazil and China sharing only 11 widespread pest thrips (Table 2 and Table 3). However, in addition to faunal differences, human perceptions and socio-economic expectations in crop production are likely to produce differing views on “pest damage” (Figure 3). Based on their individual experiences and commercial expectations, different observers will come to different conclusions as to what constitutes a pest. As with any human activity, some growers within the horticultural industry are clearly more skillful than others, often deploying a range of practices from careful selection of seedlings and planting date, to good quarantine and weed control. Such growers may produce a profitable crop requiring minimal use of chemical pest control. In contrast, a neighbouring grower may be persuaded by agricultural salesmen that the easiest approach to cultivation is to use large quantities of chemicals. In southern Australia, the citrus pest *Pezothrips kellyanus* has been shown to form mating aggregations late in the afternoon [82]. These aggregations are on exposed parts of the trees where they provide a good target for chemical sprays, but commercial pest control firms spray only in the mornings when the thrips are widely dispersed in the tree canopies. Understanding such differences in human behaviour can, at times, be equally as important as establishing the pestiferous nature of a particular thrips species. For growers, it is thus best practice to approach each pest thrips situation individually, considering the biology of that particular species on that particular crop. The alternative extreme, a semi-industrial approach with a predetermined programme of pesticide spraying, carries the risk of inducing pesticide resistance and of population resurgence. 

## Figures and Tables

**Table 1:** Thripidae species (by subfamily) impacting on human crop productivity.

**Dendrothripinae**	*Frankliniella bispinosa* (Morgan) ^2,3^
*Dendrothrips latimaculatus* Nonaka & Okajima ^1^	*Frankliniella brevicaulis* Hood ^1^
*Dendrothrips minowai* Priesner ^2^	*Frankliniella cephalica* Crawford ^1,3^
*Dendrothrips octosparsus* Wang, Mound & Tong ^1^	*Frankliniella fusca* (Hinds) ^2,3^
*Dendrothrips ornatus* (Jablonowski) ^1^	*Frankliniella gardeniae* Moulton ^1^
*Leucothrips* spp. ^1^	*Frankliniella gemina* Bagnall ^1,3^
*Pseudodendrothrips mori* (Niwa) ^1^	*Frankliniella hemerocallis* Crawford ^1^
	*Frankliniella insularis* (Franklin) ^1^
**Panchaetothripinae**	*Frankliniella intonsa* (Trybom) ^2,3^
*Anisopilothrips venustulus* (Priesner) ^1^	*Frankliniella melanommata* Williams ^1^
*Astrothrips* spp. ^1^	*Frankliniella musaeperda* Hood ^1,^
*Bradinothrips musae* (Hood) ^1^	*Frankliniella occidentalis* (Pergande) ^2,3^
*Caliothrips fasciatus* (Pergande) ^2^	*Frankliniela parvula* Hood ^1^
*Caliothrips impurus* (Priesner) ^1^	*Frankliniella schultzei* (Trybom) ^2,3^
*Caliothrips phaseoli* (Hood) ^1^	*Frankliniella tenuicornis* (Uzel) ^1^
*Caliothrips striatopterus* (Kobus) ^1^	*Frankliniella tritici* (Fitch) ^1^
*Caliothrips sudanensis* (Bagnall & Cameron) ^1^	*Frankliniella williamsi* Hood ^1^
*Dinurothrips hookeri* Hood ^1^	*Frankliniella zucchini* Nakahara & Monteiro ^1,3^
*Elixothrips brevisetis* (Bagnall) ^1^	*Fulmekiola serrata* (Kobus) ^2^
*Helionothrips aino* (Ishida) ^1^	*Kakothrips pisivorus* (Westwood) ^1^
*Helionothrips annosus* Wang ^1^	*Kurtomathrips morrilli* Moulton ^1^
*Helionothrips cephalicus* Hood ^1^	*Lefroyothrips lefroyi* (Bagnall) ^1^
*Helionothrips errans* (Williams) ^1^	*Limothrips cerealium* (Haliday) ^1^
*Heliothrips haemorrhoidalis* (Bouché) ^2^	*Megalurothrips distalis* (Karny) ^1^
*Heliothrips longisensibilis* Xie, Mound & Zhang ^1^	*Megalurothrips sjostedti* (Trybom) ^2^
*Hercinothrips bicinctus* (Bagnall) ^1^	*Megalurothrips usitatus* (Bagnall) ^2^
*Hercinothrips dimidiatus* Hood ^1^	*Microcephalothrips abdominalis* (Crawford) ^1^
*Hercinothrips femoralis* (Reuter) ^1^	*Mycterothrips glycines* (Okamoto) ^1^
*Hoodothrips lineatus* Hood ^1^	*Odontothrips loti* (Haliday) ^1^
*Panchaeothrips* spp. ^1^	*Organothrips bianchii* Hood ^1^
*Parthenothrips dracaenae* (Heeger) ^2^	*Pezothrips kellyanus* (Bagnall) ^2^
*Retithrips syriacus* (Mayet) ^2^	*Psydrothrips* spp. ^1^
*Rhipiphorothrips cruentatus* Hood ^1^	*Salpingothrips aimotofus* Kudô ^1^
*Selenothrips rubrocinctus* (Giard) ^2^	*Sciothrips cardamomi* (Ramakrishna) ^1^
	*Scirtothrips aurantii* Faure ^2^
**Sericothripinae**	*Scirtothrips bispinosus* (Bagnall) ^1^
*Neohydatothrips flavicingulus* Mirab-balou, Tong & Yang ^1^	*Scirtothrips citri* (Moulton) ^2^ *Scirtothrips dorsalis* Hood ^2,3^
*Neohydatothrips samayunkur* (Kudo) ^1^	*Scirtothrips inermis* Priesner ^1^
*Neohydatothrips burungae* (Hood) ^1^	*Scirtothrips mangiferae* Priesner ^1^
*Neohydatothrips variabilis* (Beach) ^1,3^	*Scirtothrips manihoti* (Bondar) ^1^
	*Scirtothrips perseae* Nakahara ^2^
**Thripinae**	*Stenchaetothrips biformis* (Bagnall) ^2^
*Anaphothrips obscurus* (Müller) ^2^	*Stenchaetothrips* spp. ^1^
*Anaphothrips sudanensis* Trybom ^2^	*Stenothrips graminum* Uzel ^1^
*Apterothrips apteris* (Daniel) ^1^	*Systenothrips latens* Hood ^1^
*Aptinothrips rufus* (Haliday) ^1^	*Taeniothrips euchariae* Whetzel ^1^
*Arorathrips mexicanus* (Crawford) ^1^	*Taeniothrips inconsequens* Uzel ^2^
*Aurantothrips orchidearum* (Bondar) ^1^	*Tenothrips frici* (Uzel) ^1^
*Bathrips melanicornis* (Shumsher) ^1^	*Thrips alliorum* (Priesner) ^1^
*Biltothrips minutus* (Bhatti) ^1^	*Thrips albopilosus* Uzel ^1^
*Bolacothrips* spp. ^1^	*Thrips angusticeps* Uzel ^2^
*Ceratothripoides brunneus* Bagnall ^1^	*Thrips atactus* Bhatti ^1^
*Ceratothripoides claratris* (Shumsher) ^2,3^	*Thrips australis* (Bagnall) ^1^
*Chaetanaphothrips orchidii* (Moulton) ^2^	*Thrips calcaratus* Uzel ^1^
*Chaetanaphothrips signipennis* (Bagnall) ^2^	*Thrips coloratus* Schmutz ^1^
*Chirothrips manicatus* (Haliday) ^1^	*Thrips flavus* Schrank ^1^
*Corynothrips stenopterus* Williams ^1,^	*Thrips florum* Schmutz ^1^
*Ctenothrips kwanzanensis* Takahashi ^1^	*Thrips hawaiiensis* (Morgan) ^2^
*Danothrips trifasciatus* Sakimura ^1^	*Thrips imaginis* Bagnall ^2^
*Dendrothripoides innoxius* (Karny) ^1^	*Thrips madronii* Moulton ^1^
*Diarthrothrips coffeae* Williams ^1^	*Thrips major* Uzel ^1^
*Dichromothrips corbetti* (Priesner) ^1^	*Thrips meridionalis* (Priesner) ^1^
*Dichromothrips smithi* (Zimmermann) ^1^	*Thrips nigropilosus* Uzel ^2^
*Dictyothrips betae* Uzel ^1,3^	*Thrips obscuratus* (Crawford) ^1^
*Drepanothrips reuteri* Uzel ^1^	*Thrips orientalis* (Bagnall) ^1^
*Echinothrips americanus* Morgan ^2^	*Thrips palmi* Karny ^2,3^
*Enneothrips flavens* Moulton ^2^	*Thrips parvispinus* (Karny) ^2^
*Euphysothrips subramanii* (Ramakrishna & Margabandhu) ^1^	*Thrips setosus* Moulton ^1,3^ *Thrips simplex* (Morison) ^2^
*Florithrips traegardhi* (Trybom) ^1^	*Thrips tabaci* Lindeman ^2,3^

^1^ Local or minor pest; ^2^ Frequently a pest; ^3^ Orthotospovirus vector.

**Table 2:** (**A**) Pest Thripidae species in Brazil (by subfamily). (**B**) Introduced pest Thripidae of little economic importance in Brazil.

(**A**)	
**Dendrothripinae**	*Corynothrips stenopterus* Williams ^1,5^
*Leucothrips furcatus* Hood ^1,4^	*Danothrips trifasciatus* Sakimura ^2,5^
	*Dichromothrips corbetti* (Priesner) ^2,5^
**Panchaetothripinae**	*Dendrothripoides innoxius* (Karny) ^2,5^
*Brachyurothrips anomalus* Bagnall ^2,5^	*Echinothrips americanus* Morgan ^2,5^
*Bradinothrips musae* (Hood) ^1,4^	*Echinothrips mexicanus* Moulton ^1,5^
*Caliothrips phaseoli* (Hood) ^1,4^	*Enneothrips flavens* Moulton ^1,4^
*Dinurothrips hookeri* Hood ^1,5^	*Frankliniella brevicaulis* Hood ^1,5^
*Elixothrips brevisetis* (Bagnall) ^2,4^	*Frankliniella gemina* Bagnall1,^3,5^
*Helionothrips errans* (Williams) ^2,5^	*Frankliniella musaeperda* Hood ^1,5^
*Heliothrips haemorrhoidalis* (Bouché) ^1,5^	*Frankliniella occidentalis* (Pergande) ^2,3,4^
*Heliothrips longisensibilis* Xie et al. ^1,5^	*Frankliniella insularis* (Franklin) ^1,5^
*Hoodothrips lineatus* (Hood) ^1,5^	*Frankliniella parvula* Hood ^1,5^
*Parthenothrips dracaenae* (Heeger) ^2,5^	*Frankliniella schultzei* (Trybom) ^1,3,4^
*Retithrips syriacus* Mayet ^2,5^	*Frankliniella williamsi* Hood ^1,5^
*Selenothrips rubrocinctus* (Giard) ^2,4^	*Frankliniella zucchini* Nakahara & Monteiro ^1,3,4^
	*Psydrothrips kewi* Palmer & Mound ^1,5^
**Sericothripinae**	*Scirtothrips dorsalis* Hood ^2,3,4^
*Neohydatothrips samayunkur* (Kudô) ^2,4^	*Scirtothrips manihoti* (Bondar) ^1,5^
	*Stenchaetothrips minutus* (Deventer) ^2,4^
**Thripinae**	*Thrips palmi* Karny ^2,3,4^
*Aurantothrips orchidearum* (Bondar) ^1,5^	*Thrips simplex* (Morison) ^2,5^
*Chaetanaphothrips orchidii* (Moulton) ^2,5^	*Thrips tabaci* Lindeman ^2,3,4^
(**B**)	
*Aptinothrips rufus* (Haliday)	*Leucothrips nigripennis* Reuter
*Frankliniella hemerocallis* Crawford	*Microcephalothrips abdominalis* (Crawford)
*Frankliniella tritici* (Fitch)	*Pseudodendrothrips mori* (Niwa)
*Hercinothrips bicinctus* (Bagnall)	*Thrips australis* (Bagnall)
*Hercinothrips femoralis* (Reuter)	*Thrips florum* Schmutz

^1^ Native; ^2^ Exotic; ^3^ Orthotospovirus vector; ^4^ Major pest (at least in some areas); ^5^ Minor pest.

**Table 3:** (**A**) Pest Thripidae species in China (by subfamily). (**B**) Pest Thripidae of little economic importance in China.

(**A**)	
**Dendrothripinae**	*Dichromothrips corbetti* (Priesner) ^1,5^
*Dendrothrips latimaculatus* Nonaka & Okajima ^1,5^	*Dichromothrips smithi* (Zimmermann) ^1,5^
*Dendrothrips minowai* Priesner ^1,4^	*Echinothrips americanus* Morgan ^2,4^
*Dendrothrips octosparsus* Wang, Mound & Tong ^1,5^	*Frankliniella cephalica* (Crawford DL) ^2,3,5^
*Dendrothrips ornatus* (Jablonowski) ^1,5^	*Frankliniella intonsa* (Trybom) ^1,3,4^
*Pseudodendrothrips mori* (Niwa) ^1,4^	*Frankliniella occidentalis* (Pergande) ^2,3,4^
	*Frankliniella tenuicornis* (Uzel) ^1,4^
**Panchaetothripinae**	*Fulmekiola serrata* (Kobus) ^1,5^
*Anisopilothrips venustulus* (Priesner) ^2,5^	*Lefroyothrips lefroyi* (Bagnall) ^1,5^
*Helionothrips aino* (Ishida) ^1,5^	*Megalurothrips distalis* (Karny) ^1,5^
*Helionothrips annosus* Wang ^1,5^	*Megalurothrips usitatus* (Bagnall) ^1,4^
*Helionothrips cephalicus* Hood ^1,5^	*Microcephalothrips abdominalis* (Crawford) ^2,4^
*Heliothrips haemorrhoidalis* (Bouché) ^2,5^	*Mycterothrips glycines* (Okamoto) ^1,5^
*Rhipiphorothrips cruentatus* Hood ^1,5^	*Odontothrips loti* (Haliday) ^1,5^
*Selenothrips rubrocinctus* (Giard) ^2,4^	*Salpingothrips aimotofus* Kudô ^1,5^
	*Scirtothrips dorsalis* Hood ^1,3,4^
**Sericothripinae**	*Stenchaetothrips biformis* (Bagnall) ^1,4^
*Neohydatothrips flavicingulus* Mirab-balou, Tong & Yang ^1,5^	*Stenchaetothrips* spp. ^1,5^ *Taeniothrips eucharii* (Whetzel) ^1,5^
*Neohydatothrips samayunkur* (Kudô) ^1,5^	*Thrips alliorum* (Priesner) ^1,5^
	*Thrips atactus* Bhatti ^1,5^
**Thripinae**	*Thrips australis* (Bagnall) ^2,5^
*Anaphothrips obscurus* (Müller) ^1,5^	*Thrips coloratus* Schmutz ^1,5^
*Anaphothrips sudanensis* Trybom ^2,4^	*Thrips flavus* Schrank ^1,5^
*Aptinothrips rufus* (Haliday) ^1,5^	*Thrips hawaiiensis* (Morgan) ^1,4^
*Arorathrips mexicanus* (Crawford) ^2,5^	*Thrips major* Uzel ^1,4^
*Bathrips melanicornis* (Shumsher) ^1,5^	*Thrips nigropilosus* Uzel ^1,5^
*Bolacothrips* spp. ^1,5^	*Thrips orientalis* (Bagnall) ^1,4^
*Chaetanaphothrips orchidii* (Moulton) ^1,5^	*Thrips palmi* Karny ^1,3,4^
*Chaetanaphothrips signipennis* (Bagnall) ^1,5^	*Thrips parvispinus* (Karny) ^1,5^
*Chirothrips manicatus* (Haliday) ^1,5^	*Thrips simplex* (Morison) ^2,5^
*Dendrothripoides innoxius* (Karny) ^2,5^	*Thrips tabaci* Lindeman ^1,3,4^
**(B)**	
*Apterothrips apteris* (Daniel) ^2^	*Frankliniella schultzei* (Trybom) ^2,3^
*Ceratothripoides claratris* (Shumsher) ^2,3^	*Frankliniella williamsi* Hood ^2^
*Danothrips trifasciatus* Sakimura ^2^	*Kurtomathrips morrilli* Moulton ^2^
*Dictyothrips betae* Uzel ^1,3^	*Leucothrips nigripennis* Reuter ^2^
*Drepanothrips reuteri* Uzel ^1^	*Tenothrips frici* (Uzel) ^2^

^1^ Native; ^2^ Exotic; ^3^ Orthotospovirus vector; ^4^ Major pest (at least in some areas); ^5^ Minor pest.

## Data Availability

Not applicable.
